# Fetal Pulmonary Venous Return: From Basic Research to the Clinical Value of Doppler Assessment

**DOI:** 10.1007/s00246-023-03244-4

**Published:** 2023-07-28

**Authors:** J. Portela Dias, L. Guedes-Martins

**Affiliations:** 1 Instituto de Ciências Biomédicas Abel Salazar, University of Porto, 4050-313 Porto, Portugal; 2 Departamento da Mulher e da Medicina Reprodutiva, Centro Materno Infantil do Norte, Centro Hospitalar e Universitário de Santo António, Largo da Maternidade Júlio Dinis 45, 4050-651 Porto, Portugal; 3 Unidade de Investigação e Formação – Centro Materno Infantil do Norte, 4050-651 Porto, Portugal; 4 Instituto de Investigação e Inovação em Saúde, Universidade do Porto, 4200-135 Porto, Portugal

**Keywords:** Fetal heart, Physiology, Anatomy, Fetal pulmonary venous return, Fetal echocardiography, Doppler

## Abstract

The fetal pulmonary circulation represents less than 25% of the fetal cardiac output. In comparison with the pulmonary arteries, studies on pulmonary veins are few and limited, and many questions remain to be answered. The literature reports that pulmonary veins play an important role in regulating vascular flow, forming an active segment of the pulmonary circulation. The development of more sophisticated ultrasonography technology has allowed the investigation of the extraparenchymal pulmonary veins and their waveform. The recognition of the pulmonary vein anatomy in echocardiography is important for the diagnosis of anomalous pulmonary venous connections, with a significant impact on prognosis. On the other hand, the identification of the normal pulmonary vein waveform seems to be a reliable way to study left heart function, with potential applicability in fetal and maternal pathology. Thus, the goal of this narrative review was to provide a clinically oriented perspective of the available literature on this topic.

## Introduction

Historically, the fetal venous system, and more specifically the fetal pulmonary veins, have received less attention than the arterial system. In fact, the pulmonary veins were regarded as passive conduits; however, it is now known that they are an active segment of the pulmonary circulation [1]. The fetal pulmonary circulation has specific conditions that make it unique: the collapsed lungs and the high pulmonary vascular resistance are characteristics that affect the pulmonary venous flow, making it an interesting object of study [2, 3]. Furthermore, with the introduction of high-resolution ultrasonography combined with Doppler technology, the knowledge of the normal and abnormal physiology and anatomy of the pulmonary veins has considerably increased [4, 5]. This knowledge has been applied in multiple settings, such as fetal and/or maternal pathology.

## Materials and Methods

To compose this review, a thorough literature search was repeatedly conducted in PubMed and Medline between January 2021 and February 2022, with a limitation of articles written in the English language. This literature review includes relevant articles from 1979 to 2022. The search terms used were fetal pulmonary veins, fetal pulmonary venous return, anomalous pulmonary venous return. Additionally, the references of all analyzed studies were searched to obtain necessary information.

**Fig. 1:**
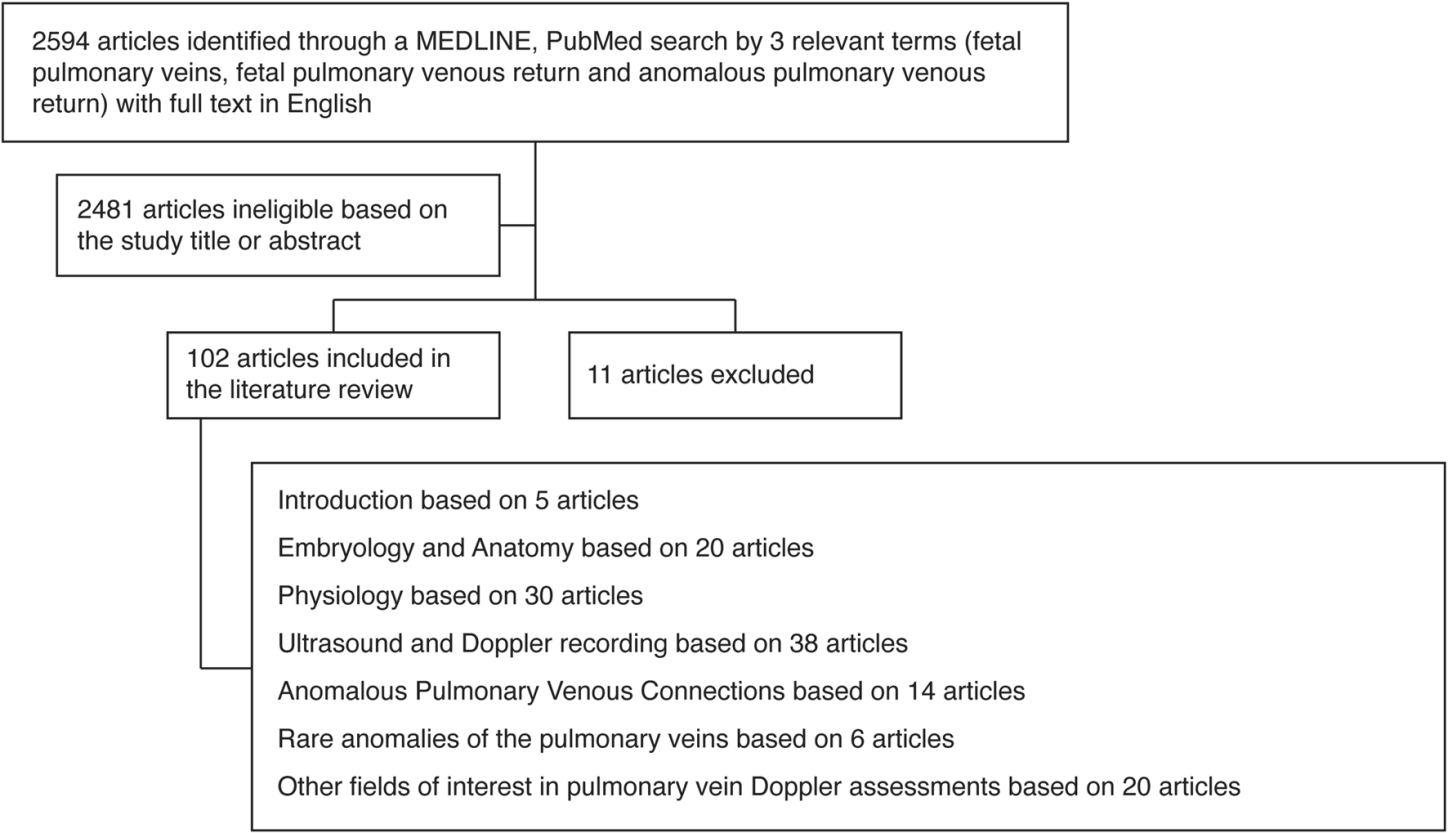
Flow diagram of the literature review

Figure 1 represents the flowchart of the search and selection process for the elaboration of this review.

## Embryology and Anatomy

A classic division of the process of lung development in humans includes five stages on the basis of histological features: the embryonic (weeks 4–7 of pregnancy), pseudoglandular (weeks 5–17), canalicular (weeks 16–26), saccular (weeks 24–38), and alveolarization stages (week 36 to infancy). The normal development of the pulmonary vasculature is an essential part of lung development and is closely correlated with airway growth [6–8].

In the developing fetal lungs, the pulmonary vasculature undergoes an initial vascular tube formation that is followed by the establishment of a hierarchical vascular system [7]. There are two basic processes that explain the formation of the pulmonary circulation: vasculogenesis and angiogenesis [6, 9–11]. Studies on humans show that the pulmonary arteries form by vasculogenesis, but less is known about the early development of the pulmonary veins [1, 12].

In the human fetus, a continuous circulation between the heart and the capillary plexus of the lungs forms as early as Day 34 of gestation, with the artery extending from the outflow tract and the vein connecting to the left atrium [1, 6, 7, 9]. With the development of the circulation, the arterial and venous pulmonary circulations become connected through a mesenchymal capillary plexus [1, 6].

Pulmonary macrovascular (arteries and veins) and microvascular (capillaries) segments arise independently and separately from the development of the left atrium. The pulmonary veins develop as part of the splanchnic venous bed [13]; CD31-labeled endothelial tubes in the mesenchyme merge on the lung bud and join to form the pulmonary venous confluence [1]. The main pulmonary veins develop as endothelial invaginations in the cranial portion of the sinus venosus during development and are later incorporated into the morphological left atrium [7, 12, 14]. The pulmonary macro- and microvascular segments are connected at the pseudoglandular stage of development [7].

In conclusion, the formation of the pulmonary veins seems to happen by vasculogenesis from the splanchnopleural mesoderm during the pseudoglandular stage, and the later growth in the intraacinar region is likely to occur by angiogenesis in the canalicular and alveolar stages [1]. These processes are genetically controlled and regulated by numerous transcription and growth factors; among these regulators, the presence of vascular endothelial growth factor (VEGF) appears to be an absolute requirement in the earliest stages of vasculogenesis, and it plays a critical role during subsequent angiogenesis [6, 7, 11].

Specific transcription factors are also the main coordinators of genetically controlled arterial-venous differentiation and patterning: the combined effect of Fox c1 and c2 and VEGF signaling defines arterial fate; the orphan nuclear receptor chicken ovalbumin upstream promoter-transcription factor II (COUP-TFII) regulates vein identity through the repression of Notch1. Hemodynamic factors are also involved in the modulation of this differentiation process [7, 15].

Other transcription and growth factors are involved in the processes of the survival and migration of endothelial cells, the regulation of vascular remodeling and the maintenance of vascular integrity (angiopoietins and their major receptor Tie-2, bone morphogenetic proteins (BMP), Fox c2, FoxO, Wnt proteins) [7, 11].

**Fig. 2:**
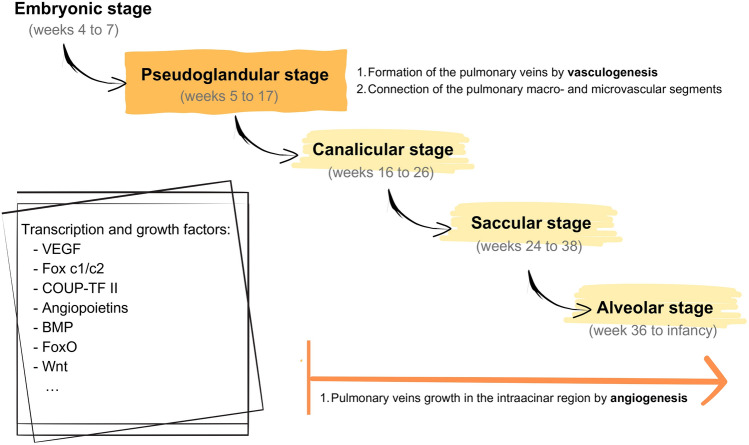
Stages of lung development: the pulmonary veins develop by vasculogenesis during the pseudoglandular stage and by angiogenesis in the subsequent stages; multiple transcription and growth factor are involved in this process

A summary of the described process is showed in Fig. 2.

**Fig. 3:**
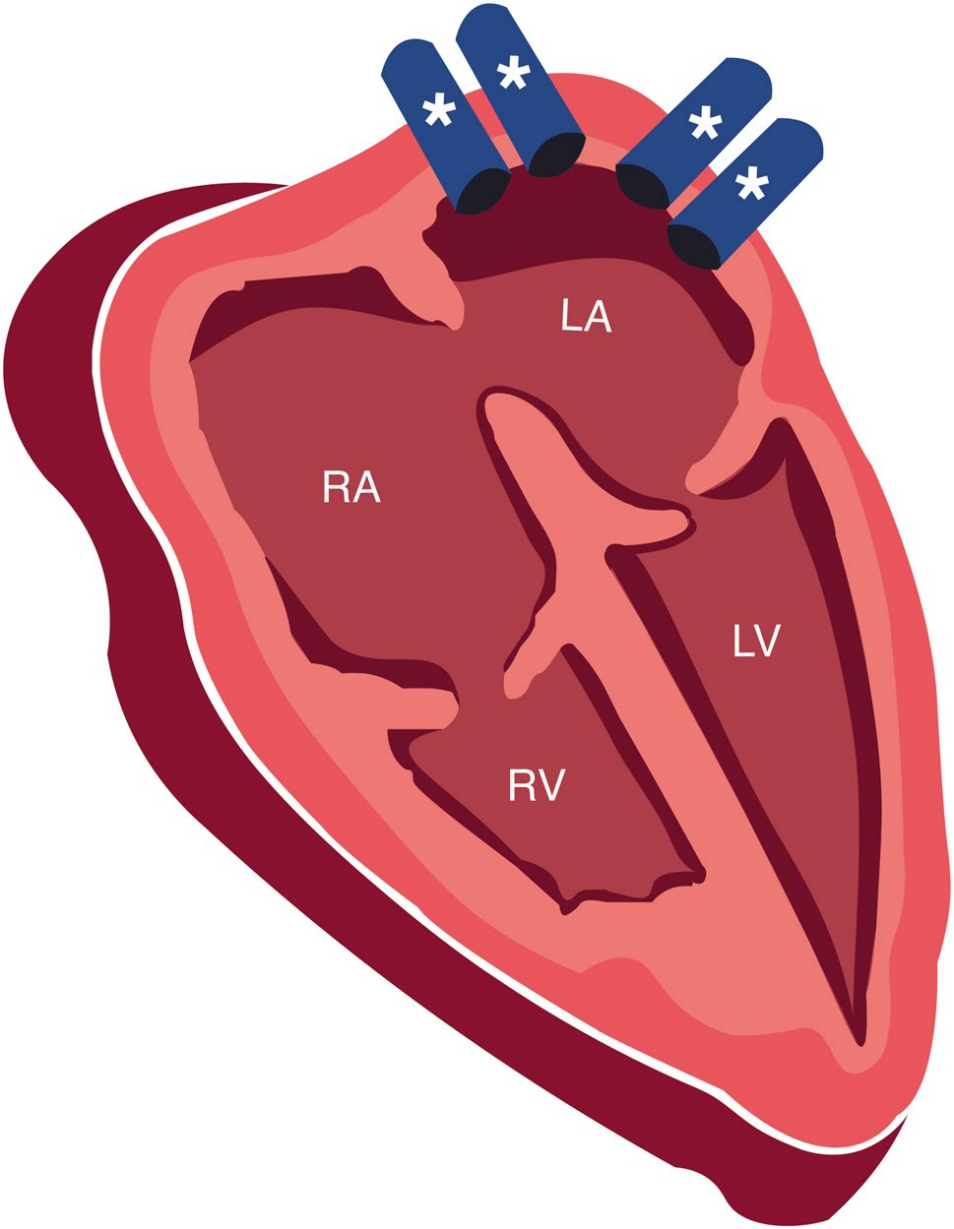
The four pulmonary veins: the pulmonary veins (*) drain the blood from the lungs, each through their own ostium in the posterior wall of the left atrium separately. *RA* right atrium; *LA* left atrium; *RV* right ventricle; *LV* left ventricle

The final result consists of an intricate microvascular pulmonary bed with the intrapulmonary part of the pulmonary veins following the intersegmental septa. At the time of birth, the branching pattern of the pulmonary circulation is similar to that observed in adults [11, 16]. The pulmonary veins leave the lungs through the pulmonary hilum, where they are located anterior and inferior to the pulmonary arteries on both sides [11, 17–19]. Normal anatomy consists of four pulmonary veins, inferior and superior on the right and left sides; these veins drain blood from the lungs (the right superior pulmonary vein drains the blood from the right superior and middle lobes, the right inferior pulmonary vein drains the blood from the right inferior lobe, the left superior pulmonary vein drains the blood from the lingula and left superior lobe of the left lung, and the left inferior pulmonary veins drain the blood from the left inferior lobe) [17, 20]. The pulmonary veins enter the posterior wall of the left atrium separately, each having its own ostium (Fig. 3) [5, 13, 21]. Other anatomical variants are possible, with most variants favoring right pulmonary venous return [19, 22].

The extrapulmonary segments of the fetal pulmonary veins are supplied by nerves extending from three epicardiac ganglionated subplexuses: the left dorsal subplexus and the left and middle dorsal subplexuses [23].

## Physiology

Fetal blood is oxygenated in the placenta and returns through the umbilical veins; it enters the right atrium and is then redirected to the left atrium through the *foramen ovale*, following its pathway to the left ventricle, whence it is pumped into the aorta. The blood received by the right ventricle is largely from the superior and distal inferior vena cava and is poorly oxygenated. Of the blood pumped out by the right ventricle, only a small portion enters the lungs via pulmonary arteries due to the high pulmonary vascular resistance (PVR); a larger portion enters the aorta via the ductus arteriosus [7, 24, 25]. In fact, compared to postnatally, the findings from Doppler studies on human fetuses in vivo suggested that the pulmonary circulation represents a small proportion of the fetal circulation: 13% at 20 weeks and 20–25% during the third trimester [13, 25, 26].

During early fetal development, the paucity of pulmonary vessels limits pulmonary blood flow; the number of fetal pulmonary vessels increases considerably between midgestation and term [27]. The increment in the blood flow to the lungs seen from 20 weeks to the third trimester is accompanied by a significant decrease in weight-indexed PVR and results mainly from the increase in the pulmonary vascular bed. However, from 30 to 38 weeks of gestation, blood flow to the lungs decreases slightly, while the weight-indexed PVR significantly increases in association with the development of fetal vasomotor tone and vasoreactivity [28]. In fact, during this gestation period, the pulmonary circulation responds to maternal hyperoxygenation with increased blood flow and decreased vascular resistance, which allows the maintenance of a high PVR by vasoconstriction in an oxygen tension-sensitive manner, preventing a striking redistribution of cardiac output from other organs to the lungs [7, 28]. The fetal pulmonary vasculature also appears to autoregulate flow through a myogenic response; this may explain why stimuli such as ductal compression, endothelium-dependent vasodilators and increased oxygen tension cause only a transient increase in fetal pulmonary blood flow [27].

In the fetus, pulmonary veins contribute a significant fraction to total pulmonary vascular resistance, regulating fluid filtration pressures in the upstream capillary network via active vasomotion [29]. Actually, the veins may play a more important role in modulating the fetal and neonatal pulmonary circulation than in adult life and show a greater sensitivity than arteries to a number of vasoconstrictor stimuli [29].

Despite the relatively hypoxic environment where the fetal lungs develop, with PO_2_ ranging from 17 to 20 mmHg, the high concentration of fetal hemoglobin, with higher oxygen affinity, allows sufficient oxygen delivery to the lungs to support their growth and metabolic functions [7, 24, 30, 31].

Vasoconstriction in response to hypoxia is a unique characteristic of pulmonary vessels, since systemic vessels relax in response to hypoxia. The effects of hypoxia are mediated by hypoxia-inducible factor (HIF) 1: under normoxic conditions, the α-subunits of HIF-1 are hydroxylated and degraded by proteosomes; under hypoxic conditions, they are stabilized. These subunits are partially responsible for the stimulation of gene transcription and the regulation of VEGF [7, 11, 32].

Most of the vasoactive mediators acting on the pulmonary circulation act via one of the cyclic nucleotides, cyclic adenosine monophosphate (cAMP) or cyclic guanosine monophosphate (cGMP); it appears that the nitric oxide-cGMP-protein kinase G (PKG) pathway may play a more important role in regulating the relaxation responses of pulmonary arteries and veins in the perinatal period than the cAMP-protein kinase A (PKA) pathway. The responses of both fetal pulmonary arteries and veins to cGMP are modulated by changes in oxygen tension (in fetal pulmonary veins, oxygen exposure increases PKG-mediated relaxation to cGMP) [29].

Taking into account their main effects on vasomotor tone, the main agents can be divided into vasodilators and vasoconstrictor modulators. A synthesis of these agents can be consulted in Table 1.

**Table 1:** Summary of the vasoactive modulators involved

Vasodilators			
*EDNO*	*PGI2* and* PGE2*	*Potassium channels*	*Calcium channels*
▪ Major endogenous agent regulating the vasodilatation of the pulmonary vasculature ▪ EDNO-dependent relaxation is greater in veins than in arteries	▪ Potent dilator properties in fetal pulmonary vessels	▪ Important regulators of vascular smooth muscle tone ▪ Activated by NO, prostacyclin and other endothelium-derived hyperpolarizing factors or by shear stress	▪ Primary pathway for Ca^2+^ influx across the plasma membrane ▪ Activated by oxygenation

Vasoconstrictors				
*Endothelins*	*PAFs*	*TxA2* and *leukotrienes*	*ROS*	*ROK*
▪ Moderate influence on vasomotor tone in fetal pulmonary vessels ▪ ET-1 is synthesized in response to shear stress, hypoxia, or ischemia ▪ ET-1 causes vasoconstriction by activating ETA receptors	▪ Significant contribution to maintaining a high tone in the fetal pulmonary circulation ▪ High circulating levels in the fetus fall after birth ▪ Upregulated by the hypoxia	▪ Potent vasoconstrictor for pulmonary veins	▪ Contribution to the increase in intracellular Ca^2+^ and subsequent contraction in pulmonary smooth muscle cells ▪ Inhibition of the production of vasodilators ▪ Upregulated in response to hypoxia	▪ Important role in the Ca^2+^ sensitization of vascular smooth muscle contraction

Other modulators		
Genetic determinants	Molecular determinants	Environmental modifiers

### Vasodilators

The vasodilators group includes endothelium-derived nitric oxide (EDNO), prostacyclin (PGI2), prostaglandin E2 (PGE2), potassium channels, and calcium channels [7].

#### Endothelium-Derived Nitric Oxide (EDNO)

EDNO is synthesized in response to a variety of stimuli and is the major endogenous agent regulating the vasodilatation of the pulmonary vasculature [7, 11, 29].

In the pulmonary vasculature, EDNO diffuses from endothelial cells into adjacent pulmonary vascular smooth muscle cells, where it causes vasodilation primarily by elevating cGMP levels [7, 27, 29]. cGMP stimulates the production of PKG, which can cause vasodilatation through direct action on myosin phosphorylation. In addition, NO can directly or indirectly activate vascular smooth muscle potassium channels, leading to hyperpolarization and a decrease in cytosolic calcium in the fetal pulmonary vasculature [27].

EDNO-dependent relaxation is greater in veins than in arteries due to the differences in the activities of endothelial cell nitric oxide synthase (eNOS), soluble guanylyl cyclase, and phosphodiesterases [29]. Additionally, the EDNO effect is limited in fetuses to maintain a high PVR, and multiple factors can influence this situation [7]. On the one hand, arginases compete with NO synthases for l-arginine as their common substrate. Two isoforms of arginase (type I and type II) are expressed in the lungs, and higher arginase expression and/or activity may contribute to lower NO production and, consequently, the maintenance of a high PVR in the fetal lungs. On the other hand, the fetal low oxygen environment can also suppress the synthesis of NO, since oxygen is a necessary substrate for its synthesis; as such, the role of EDNO in opposing pulmonary vasoconstriction is likely to be limited in the fetus [7, 33].

Beyond the decreased production of EDNO in fetal life, there is also a reduced expression of PKG protein and mRNA and a posttranscriptional modification of PKG by reactive oxygen species (ROS), with consequent decreased relaxation of pulmonary veins to cGMP in hypoxia [7].

Phosphodiesterases are responsible for degrading cGMP and may also play a role in EDNO regulation, contributing to the greater contractility of fetal pulmonary vessels, since they are abundant in lung tissues [7].

#### PGI2 and PGE2

PGI2 and PGE2 are prostanoids with potent dilator properties in fetal pulmonary vessels and are produced mainly from the endothelium. They act on vascular smooth muscle, inducing vasodilation by elevating the intracellular level of cAMP or through a direct effect on myosin phosphorylation, following the activation of adenylate cyclase [7, 11, 27, 34, 35].

Several endothelium-dependent vasodilators, including acetylcholine and bradykinin, act at least partially by enhancing prostacyclin fetal synthesis. Prostacyclin synthesis increases during the third trimester [27].

Hypoxia (PO_2_ < 50 mmHg) attenuates PGI2 production by fetal pulmonary arteries and veins [29].

#### Potassium Channels

Potassium ion channels are major determinants of cell membrane potential and are important regulators of vascular smooth muscle tone [29].

To date, at least four classes of potassium channels have been identified in the pulmonary vasculature, described based on their biophysical and pharmacological properties: the inward-rectifier (K_ir_) channel family, including the ATP-dependent channels (KATPs); the voltage-dependent (K_V_) channels; the calcium-sensitive (KCa) channels, including the BK channels; and the two-pore potassium channels [7, 36, 37]. Vascular smooth muscle cell potassium channel activation leads to the hyperpolarization of vascular smooth muscle and a decrease in cytosolic calcium, which results in vasodilatation. These channels can be activated by NO, prostacyclin, and other endothelium-derived hyperpolarizing factors or by shear stress [27].

Pulmonary veins have a coaxial structure, with a subendothelial layer of typical smooth muscle cells surrounded by a layer of cardiomyocytes, and contain K_V_, K_ir_, and BK potassium channels [7, 29]. The hypoxic fetal environment seems to result in a greater activity of BK channels [7].

#### Calcium Channels

The primary pathways for Ca^2+^ influx across the plasma membrane are voltage-operated calcium channels (VOCCs) and nonselective cation channels (NSCCs). There is some evidence that oxygenation may increase spontaneous sparking release of Ca^2+^ through ryanodine-sensitive Ca^2+^ stores, leading to the activation of BK channels, membrane hyperpolarization and, consequently, vasodilatation [7, 37].

### Vasoconstrictors

The vasoconstrictors group includes endothelins, platelet-activated factors (PAFs), thromboxane A2 (TxA2), leukotrienes, ROS and Ras human ortholog (Rho)-kinase (ROK) [7].

#### Endothelins

Endothelin 1 (ET-1) is a peptide with vasoconstrictor properties that is more potent in pulmonary veins than in arteries. It is released predominantly from endothelial cells and can bind to two receptor types, ETA and ETB receptors, which are present in smooth muscle cells [7, 11, 29].

ET-1 causes vasoconstriction by activating ETA receptors, which leads to an elevation in the intracellular concentration of Ca^2+^ and an increased sensitivity of myofilaments to Ca^2+^ [7, 27]. ET-1 may also modulate pulmonary vessel tension by stimulating the release of EDNO and prostacyclin by binding to endothelial ETB receptors; these receptors also mediate the pulmonary clearance of circulating ET-1 [7, 29, 38]. ET-1 has several other roles in the lungs, such as increasing lung endothelial cell growth factor and stimulating catecholamine release [29].

ETA receptor expression is strong throughout gestation, while ETB receptor expression is weak in the canalicular stage, increasing markedly in the second trimester [7].

When subjected to various stimuli, such as shear stress, hypoxia, or ischemia, ET-1 is transcribed, synthesized, and secreted. However, although ET-1 expression peaks at midgestation, it exerts only a moderate influence on vasomotor tone in fetal pulmonary vessels [7].

#### Platelet-Activating Factors (PAFs)

PAFs are a group of compounds synthesized from membrane lipid precursors. They possess various biological activities, including potent vasoconstrictor or relaxant activity of pulmonary vascular smooth muscle cell proliferation [7].

PAF receptor density is high in fetal lungs, and PAF contributes significantly to maintaining a high tone in the pulmonary circulation in utero [7, 29]. High circulating levels of PAF are seen in the fetus and fall after birth with the onset of oxygenation [29].

PAF synthesis by fetal pulmonary venous smooth muscle cells is more than twofold greater than that by arterial cells. This may be due to greater expression of phospholipase A2 protein and greater acetyltransferase activity in venous smooth muscle cells [29].

The hypoxic fetal environment significantly upregulates PAF-R binding and signaling, especially in fetal pulmonary venous smooth muscle, promoting PAF-mediated pulmonary vasoconstriction and the maintenance of high PVR in utero [7, 29].

PAF is inactivated by acetylhydrolase (PAF-Ah); the activity of PAF-Ah is significantly attenuated by hypoxia, suggesting that the enzymatic degradation of PAF in the fetal pulmonary vasculature is low [29].

#### Thromboxane A2 (TxA2) and Leukotrienes

TxA2 is a potent vasoconstrictor for pulmonary veins via the prostanoid receptor subtypes TP and EP1 [29, 35].

Leukotrienes are synthesized via the lipoxygenase pathway and are potent vasoconstrictors, particularly in veins. In humans, leukotrienes C4 and D4 may also play a role in EDNO-dependent relaxation of the pulmonary veins [11, 29, 39, 40].

#### Reactive Oxygen Species (ROS)

Aside from their initially described role in antimicrobial defense, ROS generation has emerged as a normal physiological response leading to essential cellular functions, such as cellular differentiation, proliferation, migration, apoptosis, and antioxidant gene expression. At the proper locations and concentrations, ROS can function as a second messenger and activate multiple signal transduction pathways within the cell. As an example, ROS, including superoxide and H_2_O_2_, activate protein kinase C-ε to regulate specific ion channels, contributing to the increase in intracellular Ca^2+^ and associated contraction in pulmonary smooth muscle cells [41].

In addition to playing an active role in modulating fetal pulmonary vasoactivity, ROS may also inhibit the production of vasodilators such as EDNO due to a posttranscriptional modification of PKG. Hypoxia may increase ROS generation from NADPH oxidase [7].

#### Rho-Kinase (ROK)

ROK is a serine/threonine protein kinase with an important role in the Ca^2+^ sensitization of vascular smooth muscle contraction and consequently with a vasoconstrictor effect in the pulmonary vasculature. In fact, increased ROK expression and/or activity in the lungs is associated with chronic hypoxia-induced pulmonary hypertension [7, 42]. High ROK activity opposes pulmonary vasodilatation in utero, contributing to the maintenance of high PVR in the fetal lungs [7].

### Other Modulators

Finally, the contribution of genetic and molecular determinants of vascular structure and function, as well as environmental modifiers, such as differences in blood flow, shear rate, vascular pressure and distension and oxygen tension, on vascular behavior of the pulmonary vasculature needs further study [27, 29].

At the end of the fetal pulmonary venous return, the extraparenchymal pulmonary veins are responsible for delivering the blood to the left atrium. These are readily distensible or collapsible vessels, with high compliance, and function as a reservoir of the left atrium, with its flow relating inversely to the left atrial pressure: when the left atrial pressure increases, the pulmonary venous flow decreases, the pulmonary veins distend, and vice versa. Their behavior at normal mean left atrial pressures also enables them to isolate the lung capillaries from the retrograde transmission of positive pressure transients from the left atrium [2, 3, 43].

At birth, the pulmonary vasculature relaxes in response to EDNO and dilator prostaglandins, and the vasomotor tone in the fetal lungs falls dramatically, allowing the lungs to assume their extrauterine gas exchange and metabolic functions [6, 27, 29, 44, 45]. These effects are oxygen dependent and modulated by PKG; the pulmonary veins are the primary sites of action of certain vasoconstrictors, such as endothelin and thromboxane [6]. The expansion of the lungs, the increased oxygen tension and the increased systemic vascular resistance contribute to a decrease in PVR [11, 27]. The pulmonary blood flow increases rapidly to 300–400 mL/min/kg body weight shortly after birth [46].

When the systemic pressure and resistance become greater than those in the pulmonary circulation, the *foramen ovale* closes; the *ductus arteriosus* begins to close within the first few hours after birth, and the shunting of blood through it decreases or ceases entirely. These important changes in the pulmonary circulation of the newborn are mainly attributable to the decrease in PVR postnatally [7].

In some pathological conditions, there is an increased synthesis of vasoactive agents that may lead to pulmonary venous constriction, increased pressures for fluid filtration and consequent formation of pulmonary edema [6].

## Ultrasound and Doppler Recording

The venous blood system is a system of low pressures and velocities that is more vulnerable to interference than the arterial blood system, and it is more difficult to access [26]. Nevertheless, the advent of high-resolution and color Doppler ultrasound has enabled a more comprehensive examination of the fetal venous system, particularly the veins at the cardiac level [5, 47]. Doppler technology allowed the establishment of reference ranges for multiple fetal vessels and their variations throughout pregnancy, as well as the establishment of associations between Doppler study alterations in these vessels and fetal pathology [48, 49]. Thus, it was possible to deepen the knowledge of the fetal heart and its hemodynamics, concluding which patterns define its normality.

Since the introduction of echocardiography, many clinical studies have demonstrated normal patterns of pulmonary venous flow in children and adults by transthoracic or transesophageal echocardiography. Pulsatile biphasic or triphasic patterns were obtained [50, 51]. However, the pulmonary venous flow pattern in fetuses and neonates has not been studied extensively [2, 44].

The fetal pulmonary veins are particularly interesting vessels to study. On the one hand, since they are connected to the left atrium, wave analysis can reflect left-sided cardiac function [26]. On the other hand, the capillary bed of the lungs and the associated venule system are subject to the same intrathoracic pressure as the atrium into which they drain, providing intrinsic intrathoracic pressure compensation during fetal breathing; moreover, the compliance of the pulmonary venule system removes remaining arterial pulsations, providing a smooth pressure at the capillary end of the veins [52]. However, the interpretation of the normal waveform has not been easy, and, to date, few studies report values and reference curves for fetal pulmonary vein waves; additionally, those that do report them are essentially studies with small samples or with poorly clarified criteria [52–54].

**Fig. 4:**
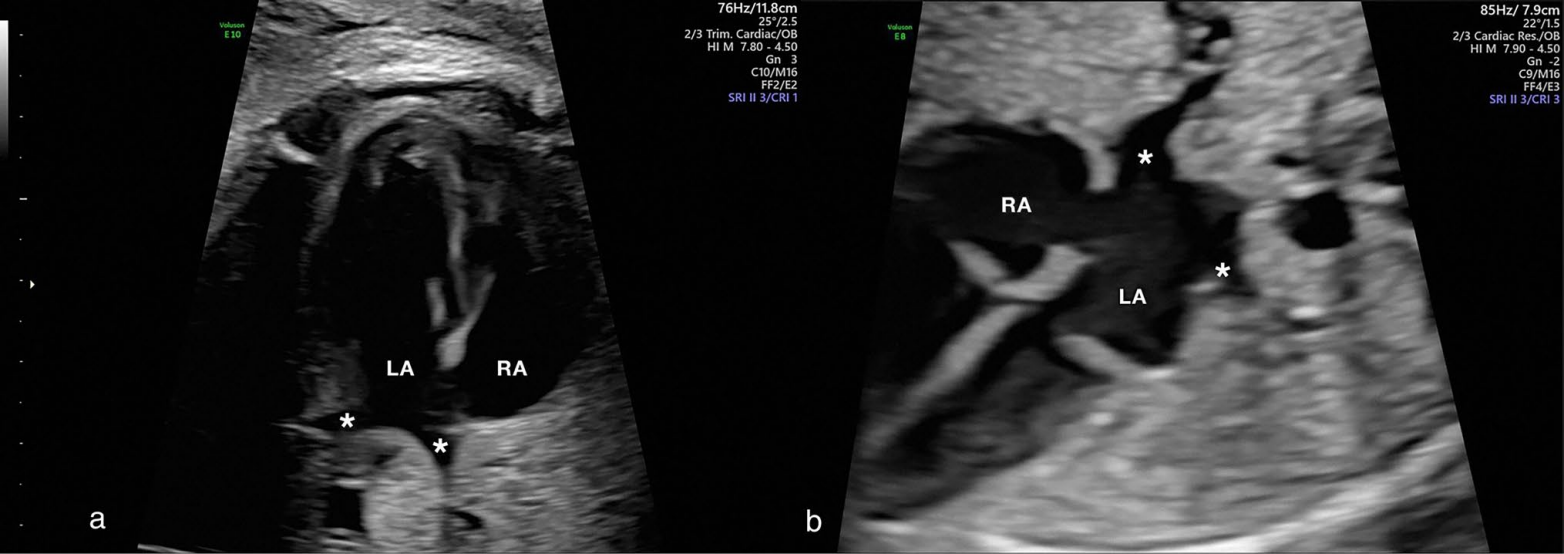
Fetal echocardiography: the ideal plane to visualize the pulmonary veins is the apical four-chamber view (**a**), showing the connection of both inferior right and left pulmonary veins (*); the pulmonary veins can be visualized in other views, as the basal or 45° four-chamber view (**b**). *RA* right atrium; *LA* left atrium

As previously mentioned, four fetal pulmonary veins drain the venous blood from both lungs to the left atrium. The ideal plane to visualize the pulmonary veins is the apical four-chamber view (Fig. 4), showing the connection of both inferior right and left pulmonary veins [5, 13, 21]. The right inferior pulmonary vein runs parallel to the interatrial septum; the left inferior pulmonary vein is perpendicular to the former, directly pointing toward the *foramen ovale* flap. The superior pulmonary veins are more difficult to visualize, requiring a slight cranial shift and angulation from the four-chamber view; however, the visualization of the two inferior pulmonary veins is usually sufficient for fetal echocardiographic evaluation unless there is a heart defect detected, when it is advisable to demonstrate at least three of these veins [5, 13, 21, 55].

**Fig. 5:**
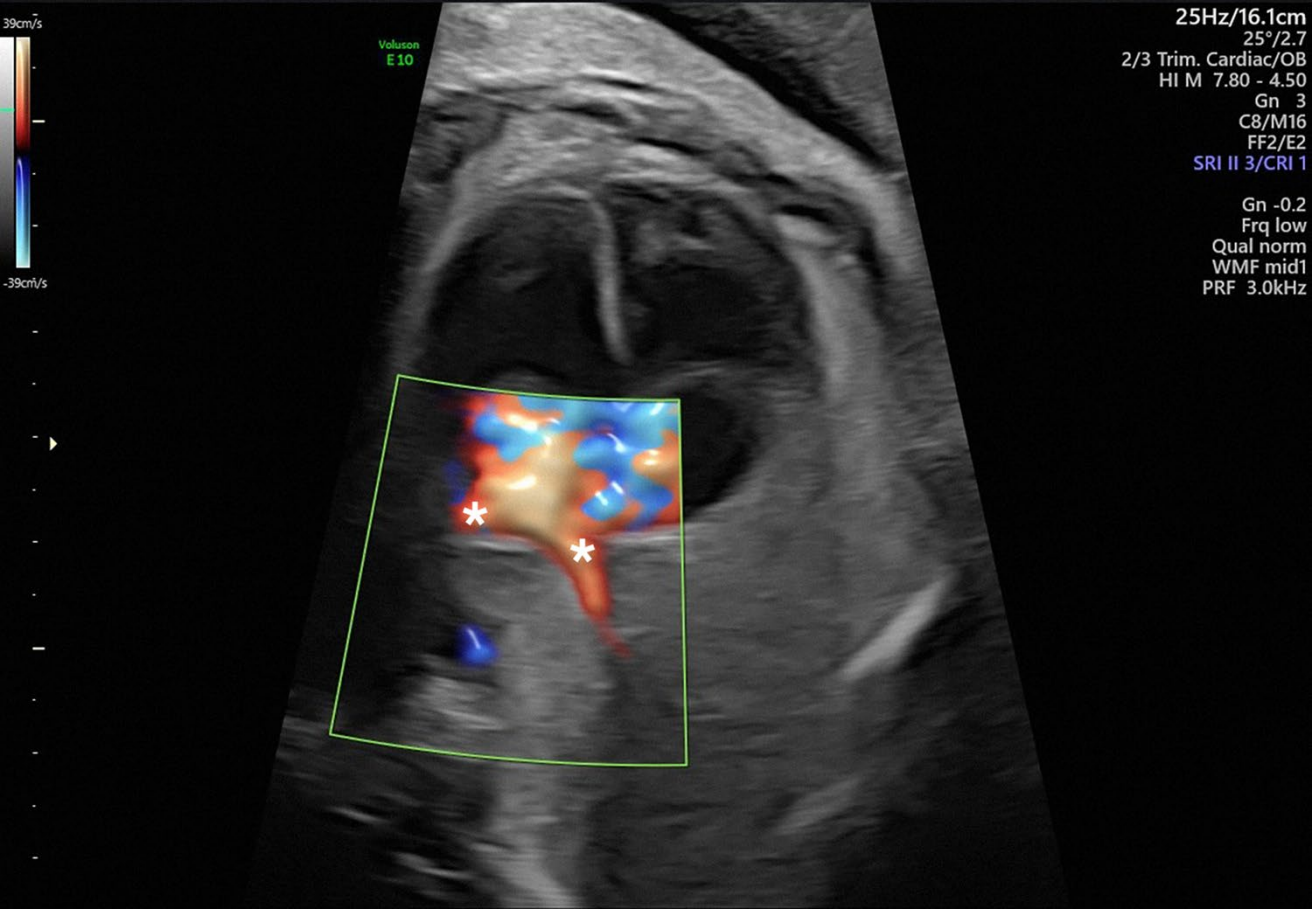
Color Doppler in fetal echocardiography: applying color Doppler can help in the identification of the pulmonary veins (*)

The identification of the pulmonary veins remains a technical challenge for some screening sonographers; as the pulmonary veins drain into the left atrium at the very posterior region of the fetal heart, it can be difficult to visualize the connection of the pulmonary veins with the left atrium by two-dimensional ultrasound. As such, color Doppler plays an important role in fetal cardiac screening examinations (Fig. 5) [5, 13, 56–58]. The basic four-chamber view combined with color velocity maps detects at least two pulmonary veins in one-third of the fetuses examined between 17 and 40 weeks of gestation. Scanning around the left atrium is an effective method to identify more pulmonary veins than the four-chamber view alone, enabling the identification of at least three pulmonary veins in approximately 95% of fetuses in this gestational period; the limitation of this technique is mainly at the end of gestation [57]. However, in the first trimester, pulmonary vein assessment remains one of the most challenging aspects of early fetal echocardiography, with under 50% of color Doppler examinations at 13 weeks of gestation providing successful pulmonary vein assessments [59, 60].

When applying color Doppler, it is important to see the color flow shoot across the back wall of the left atrium into the mid portion of the atrium to verify that the veins are not entering a confluence behind the atrium [61]. The use of new contrasting techniques, such as harmonic imaging or compound imaging, facilitates real-time visualization of the pulmonary veins [13]. Low-frequency high-definition power Doppler is a new flow imaging technique that employs an advanced compound impulse emission and is a superior technique for visualizing the filling of low-velocity blood vessels, allowing a better detection rate for the pulmonary veins; compared with traditional power Doppler, it has better axial resolution with fewer artifacts, which improves the sensitivity and can thereby aid in the detection of subtle vessels with low velocity [58, 62]. Four-dimensional (4D) sonography with spatiotemporal image correlation (STIC) is a recent technological advance that allows the acquisition of the fetal cardiac volume in a single sweep and can enhance the identification of the pulmonary veins; 4D sonography can be combined with the B-flow modality or with high-definition flow imaging (HDFI), with the latter having the advantage of being able to depict low-velocity vessels with directional information [57, 58]. In addition, the reconstructed 4D images can demonstrate the spatial relationship of the pulmonary venous confluence to the left atrium, which is useful for cardiothoracic surgeons, as can be seen in the article published in 2017 by Sun et al. [58].

To obtain the pulmonary venous waveform, spectral Doppler should be applied, with a sample size between 1.5 and 2 mm and an angle below 20–30° [2, 63]. Adjusting some technical settings, such as a low-velocity scale (velocity range should be reduced to a range of 15–25 cm/s) or pulse repetition frequency (filters of 50–100 Hz), may improve the visualization of the pulmonary veins [5, 13, 57, 63, 64].

Laudy et al. [65] studied the fetal pulmonary veins of 123 pregnant women with gestational ages between 20 and 40 weeks, with an 81% success rate. Doppler study of the fetal pulmonary veins showed a biphasic wave, with a systolic and diastolic component and with an anterograde flow (constant flow throughout the cardiac cycle, toward the left atrium). In this study, there were no significant changes between the diastolic and systolic peaks in the pulmonary veins; however, there was a significant increase in the systolic and diastolic peaks with increasing gestational age. Further studies confirmed these findings [2, 66–69]. Similar features were observed in the Doppler waves obtained in more proximal or distal portions of the pulmonary veins [67–69].

**Fig. 6:**
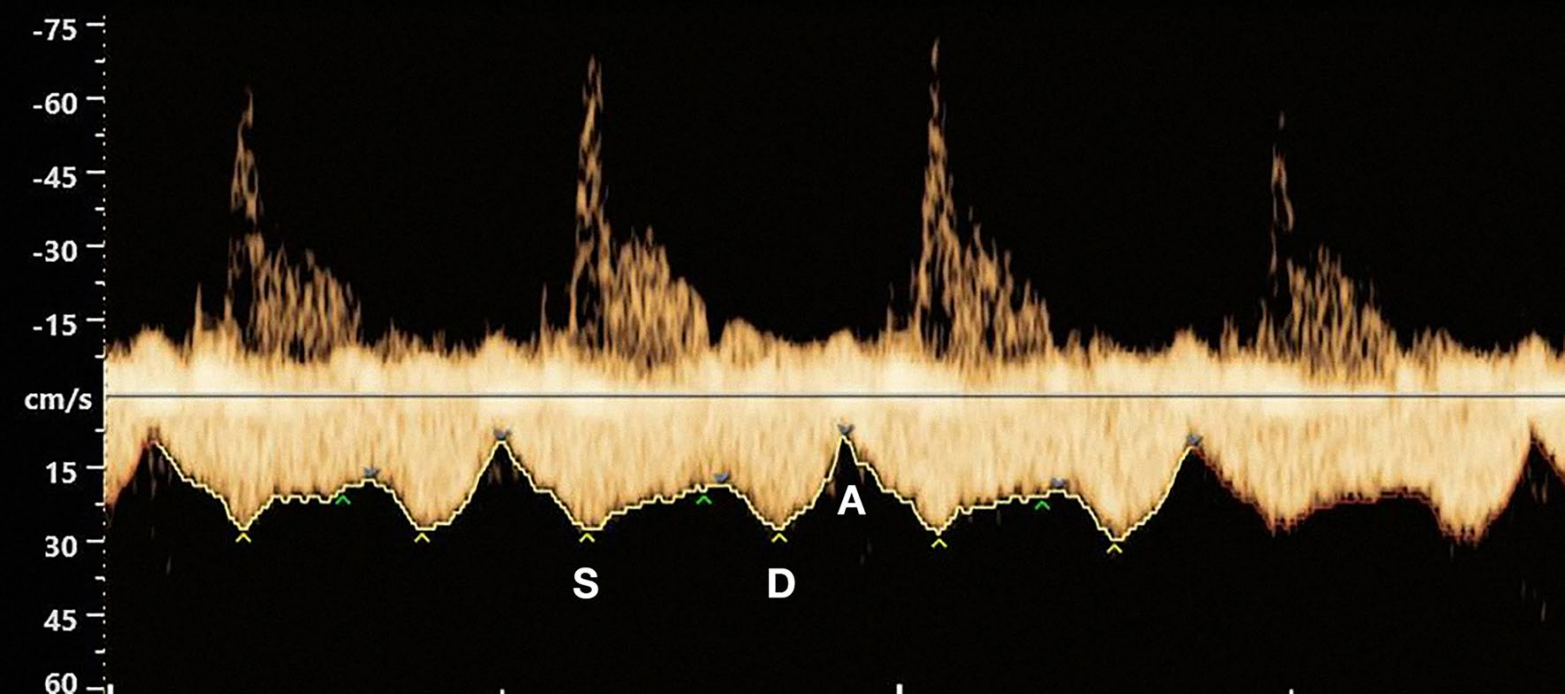
Fetal pulmonary veins’ waveform: the normal waveform of the fetal pulmonary veins is characterized by a pulsatile flow: S-wave, during ventricular systole (S); D-wave, during early ventricular diastole (D); A-wave, a nadir at the end of diastole (A); the simultaneous recording of Doppler waveforms from pulmonary artery allows the qualitative assessment of the relationship of atrial to ventricular contraction

As in adults, the fetal pulmonary venous flow patterns have also been demonstrated to be influenced by dynamic changes in left atrial pressure created by the contraction and relaxation of the atrium and ventricle [2, 3, 13, 51, 70–72]. As such, the normal waveform of the fetal pulmonary veins is characterized by a pulsatile flow [2, 26, 44, 66, 67, 70, 73, 74] (Fig. 6):

- S-wave, during ventricular systole;
- D-wave, during early ventricular diastole;
- A-wave, a nadir at the end of diastole (during atrial contraction).

The systolic peak reflects the suction effect caused by atrial relaxation and by the downward movement of the mitral valve during ventricular systole. Sometimes, these two events are dissociated, resulting in a double systolic peak. The diastolic peak mirrors the rapid emptying of the left atrium during ventricular relaxation. The A-wave represents the ventricle filling pressure during atrial contraction; in the fetus, the flow at the end of diastole is usually a low but positive flow [13, 43, 67, 70].

The fetal pulmonary vein waveform is therefore very similar to that of the ductus venosus, with a forward triphasic flow throughout the heart cycle [5, 13, 71]. Additionally, the flow pattern of fetal pulmonary veins is similar to that found in children and adults. However, in adults, the systolic velocity is consistently higher than the diastolic velocity, and the A-wave usually presents a retrograde flow, in contrast to the anterograde flow throughout the cardiac cycle seen in most fetal assessments [2, 67, 72]. These changes seem to be due to hemodynamic changes that occur postnatally after the closure of the *foramen ovale*, which lead to significant changes in the left atrium pressures and in the stiffness of the heart walls [67, 72]. On the other hand, the extraparenchymal pulmonary venous system in the fetus shows limited reservoir function because the fetal lungs are collapsed. When a small reservoir is combined with elevated pulmonary vascular resistance, the result will be an extraparenchymal venous pressure higher than that in the left atrium; this can explain the absence of retrograde atrial flow in the fetal pulmonary veins [2, 72].

After identifying the usual wave pattern of the fetal pulmonary veins, an attempt was made to define reference ranges. The first studies focused essentially on the values of the peak velocities of the S-, D-, and A-waves [2, 65–67, 72, 73]. In 2019, a study included 184 healthy fetuses, from 18 to 39 weeks, confirming a steady increase of the maximal velocity of fetal pulmonary venous blood flow and establishing a regression equation for the maximal velocity as a function of gestational age in days: *V*_max_ (cm/s) = 0.1 × GA (in days) + 5.5 (*r* = 0.45, CI 0.95) [75].

The pulmonary vein pulsatility index (PI) [peak velocity (systolic or diastolic) minus presystolic velocity/mean velocity] reflects the relative impedance to the forward flow and seems to be more comparable than absolute values of individual waveforms and independent of the insonation angle [12, 64]. Lenz et al. [53] tried to establish reference ranges for the pulmonary vein PI and peak velocity index for the veins (PVIV). After confirming the intraobserver reproducibility, a significant increase in systolic and diastolic peak velocities, as well as in the end-diastolic velocity, was obtained throughout the second half of pregnancy, which is compatible with the increased pulmonary blood flow in this period. In contrast, the S/D ratio did not change significantly with gestation. The PI and the PVIV decreased with gestational age, perhaps due to the increased left atrial and ventricular compliance and better emptying of the left atrium (since the systolic and diastolic peak velocities increase proportionally throughout the second half of gestation, this decrease in pulsatility may be due to an exponential increase in the A-wave). A more recent study [54] included 365 fetuses between 18 and 42 weeks of gestation and had similar results, with a decrease in the pulmonary vein PI with gestational age.

Zielinsky et al. [12] conducted an experimental hemodynamic study that showed that the pulmonary vein pressure varied depending on the recording site, with the pulmonary vein diameter at the different sites probably being the main determinant. Indeed, in the normal fetus, the pulsatility of the pulmonary vein decreases along the way from the lungs to the heart, and this parameter is inversely correlated with the cross-sectional diameter of the pulmonary vein, which increases from the proximal to the distal portion of the vessel.

It is also believed that fetal breathing movements impact pulmonary venous blood flow. In apnea, the pressure of intrathoracic organs on the fetal heart limits ventricular distensibility, particularly as a result of nonexpanded lungs. During fetal breathing movements, the fall in intrathoracic pressure leads to an improvement in the compliance and filling pressure of the left ventricle, which reflects changes in venous return and its end-diastolic volume. In line with this, a study with 22 fetuses with gestational ages between 25 and 34 weeks [69] showed a significant reduction in the pulmonary vein PI during fetal breathing movements. The anterograde presystolic wave of pulmonary venous flow showed a significantly increased velocity during breathing movements, probably due to a reduction in the left atrial pressure.

Technological progress has significantly improved the visualization and assessment of the pulmonary veins. Dong et al. [56] studied the pulmonary veins of fetuses between 12 and 40 weeks, with a detection rate of 75% of the four pulmonary veins using the enhanced-flow technique; images of the application of this technique can be consulted in the original article. The presence of an inverted A-wave occurred in only 3.3% of the fetuses, and the peak velocities were higher in the right pulmonary veins, which may be due to the larger size of the right lung and the consequent need for greater blood flow for its development.

It is now generally accepted that the pattern of pulmonary venous blood flow is determined by left atrial pressure changes [70]. However, despite significant advances in the study of fetal pulmonary veins, many questions remain to be answered. The few studies that report values and reference curves for fetal pulmonary vein waves present small samples and/or unclear criteria.

## Anomalous Pulmonary Venous Connections

An anomalous pulmonary venous connection is the failure of the common pulmonary vein and its splanchnic components to meet. The postnatal hemodynamic consequence of anomalous pulmonary venous connection is that oxygenated blood from the lungs reaches directly or indirectly (together with the systemic veins) into the right instead of the left atrium. If arterialized blood does not reach the left atrium, the neonate may present with cyanosis [13]. Anomalous pulmonary venous connections may be separated into two subgroups: partial (PAPVCs) and total (TAPVCs) [4, 13, 74].

TAPVCs represent a rare form of congenital heart disease in which all the pulmonary veins connect directly or indirectly to the right atrium [4, 5, 13, 58, 74, 76]. A TAPVC as an isolated finding is present in 1 per 17,000 live births, and approximately one-third of patients have an associated major anomaly (*cor biloculare*, single ventricle, *truncus arteriosus*, the transposition of the great arteries, pulmonary atresia, coarctation, a hypoplastic left ventricle, anomalies of the systemic veins); the remaining two-thirds have isolated TAPVCs [4]. A family history of TAPVCs might increase the risk of recurrence, which suggests, at least in part, a genetic cause for the disease [76].

In PAPVCs, there is a failure of one, two or three pulmonary veins to connect to the left atrium; these veins remain connected to a systemic vein—the superior cava, the brachiocephalic vein or the coronary sinus [4, 5, 13, 74]. Usually, these malformations are also accompanied by an atrial septal defect [4], and the right pulmonary veins are more often involved than the left [5].

**Fig. 7:**
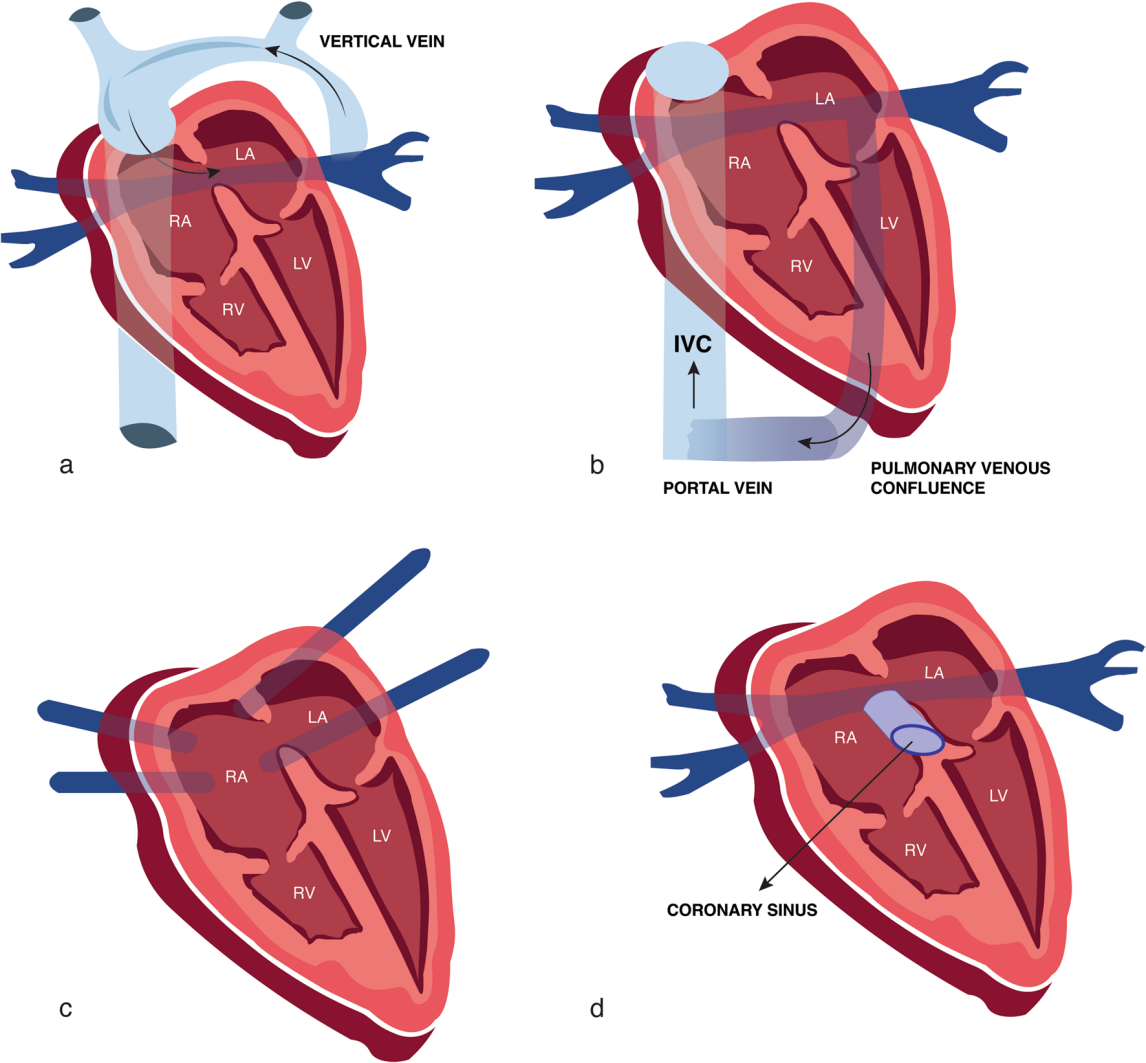
Variations of TAPVCs: supracardiac (the pulmonary veins connect to a common confluent vein behind the heart, draining via an ascending vertical vein to the brachiocephalic vein and superior vena cava—**a**, infracardiac (the pulmonary veins connect to a common confluent vein, which drains via a descending vertical vein crossing the diaphragm into the portal veins in most cases—**b**, cardiac (the pulmonary veins connect directly to the right atrium—**c** or indirectly to a dilated coronary sinus, which then drains the blood to the right atrium—**d**. *RA* right atrium; *LA* left atrium; *RV* right ventricle; *LV* left ventricle; *IVC* inferior vena cava

Variations of TAPVCs and PAPVCs are common, but generally, four types of connections can be distinguished (Fig. 7): supracardiac (44%; the pulmonary veins connect to a common confluent vein behind the heart, draining via an ascending vertical vein to the brachiocephalic vein and superior vena cava), infracardiac (26%; the pulmonary veins connect to a common confluent vein, which drains via a descending vertical vein crossing the diaphragm into the portal veins in most cases but occasionally into the hepatic veins or into the ductus venosus), cardiac (21%; the pulmonary veins connect directly to the right atrium or indirectly to a dilated coronary sinus, which then drains the blood to the right atrium) and mixed connections (9%) [5, 13, 21, 74]. An alternative classification system divides TAPVCs into two groups: supradiaphragmatic TAPVCs without pulmonary venous obstruction and infradiaphragmatic TAPVCs with pulmonary venous obstruction (at the interatrial septum or intrinsic/extrinsic to the anomalous venous channel) [4].

Total or partial anomalous pulmonary venous connections occur in 2% of live births [5, 13] and can occur as an isolated finding or as part of complex heart anomalies, mainly right atrial isomerism (asplenia syndrome), which is found in more than 75% of cases [5, 13, 76–78]. Despite the effort to include the visualization of at least one or two pulmonary veins in routine screenings, the diagnosis of TAPVCs has been reported prenatally only in case reports or very small series, being commonly a missed prenatal diagnosis [13, 77, 79, 80]. In a 6-year study, only eight (2.4%) of 331 cases of TAPVCs born had an accurate prenatal diagnosis, and the median gestational age at which TAPVCs were diagnosed was relatively late, at almost 27 weeks [76].

**Fig. 8:**
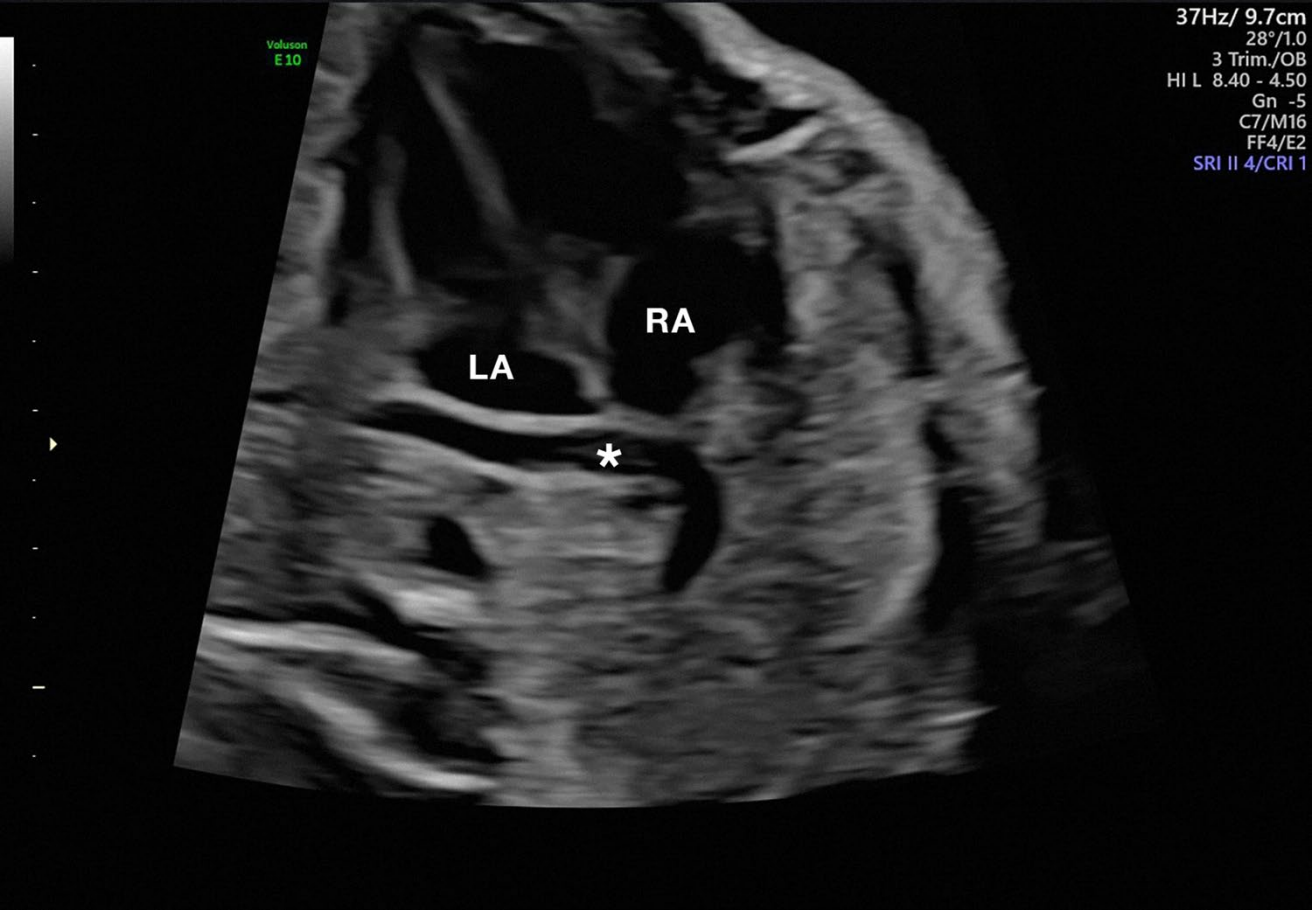
Ultrasound image of supracardiac TAPVC: the pulmonary veins connect to a common confluent vein behind the heart (*), draining via a vertical vein to the brachiocephalic vein and superior vena cava. *RA* right atrium; *LA* left atrium

In fact, it can be very difficult to visualize the lack of connection of the pulmonary veins with the left atrium [5, 13, 58, 76, 80]. Predictably, fetal echocardiography has a higher rate of correct diagnosis than routine screening [61]. One subtle sign could be a narrow left ventricle (in comparison to the right ventricle) due to the lack of pulmonary venous return into the left ventricle [5, 13], and in TAPVCs, a right ventricle volume overload, an enlarged right atrium, and a bowing leftward of the interatrial septum will be noted [4]. However, this disproportion of the right and left heart may become obvious only as pulmonary blood flow increases in the latter stage of pregnancy; the presence of a large atrial septal defect may also prevent this disproportion because it allows some of the excessive venous return in the right atrium to reach the left atrium [61, 76, 77, 80]. In fact, the four-chamber view is usually normal at 20 weeks’ gestation; an exception to this is TAPVCs to the coronary sinus, where a dilated coronary sinus can be seen at 18–20 weeks [13, 76]. One of the first hints for the diagnosis of TAPVCs seems to be the demonstration of a confluent vein behind the left atrium (Fig. 8). On axial images through the fetal chest, the space between the left atrium and the spine should contain only the descending aorta, usually located left of, and anterior to, the spine; the presence of an additional vessel in the retrocardiac space raises the suspicion of an anomalous pulmonary venous connection [61, 77, 78]. A supracardiac TAPVC can be suspected by detecting a dilated brachiocephalic vein (since in addition to blood coming from the left side of the upper extremity, blood flow from the lungs will pass through it) or by visualizing a left persistent superior vena cava as a fourth vessel in the three-vessel-trachea view with upward flow toward the upper thorax (unlike in isolated left persistent superior vena cava), while in the infracardiac type, a vertical, long, and thin vein crossing the diaphragm into the liver can rarely be demonstrated using color Doppler [5, 13, 61, 76]. Another possible sign of concern for a lack of pulmonary vein attachment is the absence of the Coumadin ridge, a normal pericardial reflection or infolding of tissue between the left atrial appendage and the left pulmonary vein, which extends a short distance medially, forming the posterolateral aspect of the left atrium; the presence of a Coumadin ridge can therefore be helpful in ruling out TAPVCs [61].

Pulsed Doppler can also assist in the diagnosis: flow pulsations are decreased or absent in TAPVCs with an infradiaphragmatic connection or show an atypical pattern resembling the inferior vena cava or a more dumped pattern in cardiac or supracardiac connections [13]. Three-dimensional spatiotemporal image correlation (STIC) fetal echocardiography has also been shown to be of incremental value in further evaluation of the classification of TAPVC types and the imaging of TAPVC drainage pathways [81].

Cases of PAPVCs are even more difficult to detect; Doppler flow studies and color mapping are indispensable, and a finding of right ventricular volume overload with an intact atrial septum may mean that a PAPVC anomaly is present [4, 13]. In one special group of PAPVCs called Scimitar syndrome, there is a combination of cardiopulmonary malformations characterized by the presence of anomalous pulmonary venous return from the right pulmonary vein(s) to the inferior vena cava, resulting in right lung hypoplasia; in these cases, although prenatal diagnosis is uncommon, the detection of a shifting of the heart into the right thorax and the visualization of the connection of the right pulmonary vein(s) into the inferior vena cava in a longitudinal plane (using color Doppler) can raise suspicion for this syndrome [5, 82].

The clinical presentation and prognosis in cases of PAPVCs and TAPVCs depend on the number of pulmonary veins involved, site of anomalous connection, presence of pulmonary venous obstruction, presence and size of the interatrial connection and presence of additional cardiac or extracardiac defects [4, 5, 77]. If detected and corrected surgically early in life, isolated TAPVCs may have a good outcome; however, the prognosis is poor when there is an association with other cardiac anomalies, such as right atrial isomerism and pulmonary venous obstruction. In cases of TAPVCs with an obstruction in the anomalous venous channel, the patient’s condition usually deteriorates rapidly after birth, with severe respiratory distress and cyanosis; the neonate typically dies in the 1st weeks of life [4, 5, 77, 83]. If a TAPVC is suspected, echocardiography should be performed soon after birth to ensure adequate atrial shunting, or an atrial septostomy may be needed [61]. In infradiaphragmatic TAPVCs, a significant obstruction may develop only after birth as the pulmonary venous flow increases and the ductus venosus constricts [77]. On the other extreme, some PAPVCs may not even show clinical signs in the neonate or the infant and are detected only accidentally [5].

As ultrasonography equipment becomes more sophisticated with higher resolution, prenatal detection of isolated anomalous pulmonary venous connections may improve. Increased training and awareness are fundamental because the accuracy of antenatal diagnosis relies largely on the effectiveness of fetal anomaly screening in obstetric, rather than cardiac, units.

## Rare Anomalies of the Pulmonary Veins

Other rare anomalies of the pulmonary venous system include the presence of arteriovenous fistulae within the lungs, with some cases reported leading to volume overload. A hint for the diagnosis was the identification of sonolucent echoes in the lungs with a high turbulent flow on color Doppler [13, 84–86]. Another rare condition is the direct connection of the pulmonary artery branch directly to the left atrium bypassing the vein [13, 87, 88].

## Other Fields of Interest in Pulmonary Vein Doppler Assessments

More recently, there has been particular interest in the study of the pulmonary veins to identify fetal pathology [54, 89, 90]. In fact, the A-wave observed in various sections of the venous system has proven to be an important clinical marker that reflects alterations in cardiac function; a high amplitude of this wave can be generated in the fetal atrium as a result of Frank–Starling mechanisms when the atrium is exceptionally distended during bradycardia (e.g., atrioventricular block) or when the atrial contraction comes at a time when the atrioventricular valves are closed (e.g., tachycardia) or during adrenergic drives [26].

In the fetus, a maximum of 25% of cardiac output passes through the lungs, and the main blood flow into the left atrium passes through the *foramen ovale*. Given that the fetal pulmonary vein waveform reflects changes in the left atrium, the determinants for the pattern seen include pulmonary volume flow, foramen ovale size and flow, left atrial compliance, atrial contraction (end-diastolic pressure), left ventricular size and performance, and mitral valve size and function [13, 26]. In fact, a patent mitral valve in normal fetal hearts or a wide interatrial connection in obstructive left ventricular disease are associated with a relatively low pulsatility in the pulmonary venous flow velocity waveform. Additionally, in cases of left heart obstruction with a patent but restrictive *foramen ovale*, the pressure in the left atrium is increased during atrial contraction, leading to a reversed flow in the fetal pulmonary veins. A closed *foramen ovale* in hypoplastic heart syndrome is the extreme form of increased left atrial pressure in the fetus [70]. In fact, the degree of atrial obstruction in hypoplastic left heart syndrome can be predicted by the amount of A-wave reversal in the pulmonary veins; the most severe form is consistent with reversed flow, which is superior to anterograde flow or when the flow is to and through [5, 70, 91–93].

Schenone et al. [90] concluded that the identification of inverted A-waves in the pulmonary veins in the first trimester ultrasound, in combination with nuchal translucency, tricuspid insufficiency and ductus venosus inversion, could increase the detection rate of fetal heart disease to 83.3%.

The use of pulmonary vein PI in fetuses has been proposed as a good echocardiographic Doppler parameter to assess fetal cardiac dynamics since it seems to reflect left atrial dynamics and is a reproducible measurement [63, 64]. As such, theoretically, if the compliance of the left ventricle is reduced, the presystolic component (A-wave) of the pulmonary vein flow will be decreased, absent or reversed, with a consequent increase in PI, representing the increased impedance to left atrial inflow [64].

The association of pulmonary venous flow with systolic and diastolic cardiac times assessed at the level of the mitral valve is used in cardiology to assess diastolic function. This association was analyzed in fetuses, and pulmonary venous inflow into the left atrium occurred predominantly during the filling and ejection phases of the cardiac cycle. The cardiac diastolic and systolic time intervals as well as the distribution of pulmonary venous flow velocity integrals between these cardiac time intervals remained unchanged with advancing gestational age [66].

Interestingly, a case report of congenital lobar emphysema [94] described the absence of physiological increase in the peak systolic velocity of the pulmonary veins from 23 to 38 weeks; this observation is supported by animal experiments suggesting a significant reduction in pulmonary vascular development in congenital lobar emphysema cases. As such, the authors propose the systematic evaluation of the pulmonary venous flow on examination of the fetus affected by echogenic lung lesions.

Pulmonary venous flow was also relevant for the development of a technique for the evaluation of fetal arrhythmia, which consists of the simultaneous recording of the intraparenchymal pulmonary artery and vein. Since both vessels are adjacent to each other within the lungs, showing opposite flow directions, simultaneous spectral Doppler sampling will demonstrate waveforms on both sides of the baseline: the peak of the pulmonary artery reflects ventricular systole, whereas atrial contraction is identified by a nadir in venous flow. Using this technique, the examiner can qualitatively assess the relationship of atrial to ventricular contraction and quantify the arteriovenous time [95].

Functional echocardiography, including the assessment of pulmonary vein blood flow, has also been employed in fetuses with extracardiac conditions, such as fetal growth restriction (FGR), and has been proven to provide important information for clinical management decisions [63].

FGR is characterized by changes in fetal hemodynamics as a result of the alterations in preload and afterload, ventricular compliance and myocardial contractility that occur to protect vital organs from hypoxia. Doppler examinations are often used to accurately assess the extent of deterioration in these fetuses. With progressing fetal deterioration, the S/D ratio of the umbilical artery velocity wave increases, evolving posteriorly to an absent or reversed end-diastolic flow in the umbilical artery; alterations in the aortic isthmus and middle cerebral artery flow can occur, as well as decreased A-waves in the *ductus venosus* [96]. In placental insufficiency, the heart plays an important role in managing the progressive adaptative changes to protect fetal vital organs. The increased uteroplacental vascular impedance seen contributes to preferential flow to the left ventricle, which leads to an alteration of its compliance and subsequent increased left atrial pressure. Pulmonary vein PI is a good parameter to assess atrial dynamics that can reflect the increased impedance to left atrial filling [63]. In fact, fetuses with FGR show lower A-wave velocity and an increased pulmonary vein PI, reflecting some degree of left ventricular dysfunction, which usually precedes right ventricle functional abnormalities [63, 97]. In other words, subclinical left heart dysfunction occurs from an early stage of fetal deterioration [96]. As such, the pulmonary vein PI could be used as an early predictor of cardiac dysfunction in FGR before the expected late increase in *ductus venosus* impedance and could also be useful as a predictor of perinatal mortality [63, 97]. Furthermore, as FGR progresses, the resultant fetal hypoxia can cause cell damage, which could ultimately be documented by a reverse pulmonary vein A-wave, similar to the Doppler pattern flow of *ductus venosus* [63]. Similar conclusions have been found in fetuses with placental insufficiency-induced oligohydramnios, with an increased pulmonary vein PI when compared to a control group [96].

Taking these findings into consideration, it seems reasonable to consider the potential implications of the pulmonary vein PI in other maternal conditions that may be associated with fetal cardiac or hemodynamic changes.

Maternal diabetes is a known risk factor for fetal myocardial hypertrophy, which may be found in up to 35% newborns from affected mothers. Significant changes in cardiovascular flow are seen in fetuses from diabetic mothers, especially in pregnancies with inadequate glycemic control. Zielinsky et al. [98] demonstrated that fetuses from diabetic mothers have a higher pulmonary vein PI than fetuses from mothers with normal glycemia. This could be due to fetal left ventricular hypertrophy and consequent decreased ventricular compliance; the increase in left atrial pressure leads to a restriction of pulmonary venous emptying, resulting in a decrease in presystolic velocity in the pulmonary vein or reverse flow in presystole. However, a prospective case‒control study [99] did not confirm these findings, since it did not show any differences in the pulmonary vein PI throughout pregnancy between diabetic and nondiabetic mothers.

Similarly, a case‒control study [100] did not find any significant differences in pulmonary vein PI between fetuses from mothers with and without intrahepatic cholestasis of pregnancy.

Preterm premature rupture of membranes (PPROM) can induce fetal inflammatory response syndrome (FIRS). The fetal myocardium is a potential target organ of proinflammatory cytokines released during FIRS, and it was hypothesized that changes in the pulmonary vein Doppler waveform could help in the assessment of diastolic cardiac function. In fact, Romero et al. [101] obtained significantly higher S, D, and A velocities in fetuses with preterm PROM than in those from a control group, which may be due to a lower impedance to blood entering the left atrium during systole and early diastole and may also be associated with a more compliant left ventricle.

## Conclusion

The investigations done in the last decades in the field of fetal pulmonary venous return allowed a better understanding of the anatomy, embryology, and physiology of the fetal pulmonary veins. It is now generally accepted that the pulmonary venous system has an active role in the maintenance of high pulmonary vascular resistance.

It is also accepted that, for its intrinsic characteristics, the extraparenchymal pulmonary veins represent Doppler-accessible key vessels for the study of the left atrium and cardiac function, with potential applicability in fetal pathology, such as fetal growth restriction.

Despite the great evolution described in the knowledge of the pulmonary venous system, there are still some questions to be answered. The lack of reference curves for the velocities and indices of the pulmonary vein waveform makes it more difficult to establish associations with fetal or maternal pathology. Additionally, the diagnosis of anomalous pulmonary venous connections is still rare prenatally, which shows a need to reinforce sonographers’ knowledge about the importance of the visualization of the pulmonary veins.
