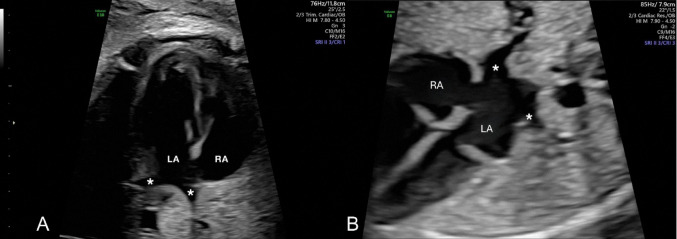# Correction: Fetal Pulmonary Venous Return: From Basic Research to the Clinical Value of Doppler Assessment

**DOI:** 10.1007/s00246-026-04175-6

**Published:** 2026-02-11

**Authors:** J. Portela Dias, L. Guedes‑Martins

**Affiliations:** 1 Instituto de Ciências Biomédicas Abel Salazar, Universityof Porto, 4050‑313 Porto, Portugal; 2 Departamento da Mulher e da Medicina Reprodutiva, CentroMaterno Infantil do Norte, Centro Hospitalar e Universitáriode Santo António, Largo da Maternidade Júlio Dinis 45, 4050‑651 Porto, Portugal; 3 Unidade de Investigação e Formação – Centro MaternoInfantil do Norte, 4050‑651 Porto, Portugal; 4 Instituto de Investigação e Inovação em Saúde, Universidadedo Porto, 4200‑135 Porto, Portugal

**Correction to: Pediatric Cardiology (2023) 44:1419–1437**

10.1007/s00246-023-03244-4

In this article, Figure 4b appeared incorrectly and have now been corrected in the original publication. For completeness and transparency, the old incorrect version and corrected version are displayed below.

Incorrect Fig. 4

**Figure:**
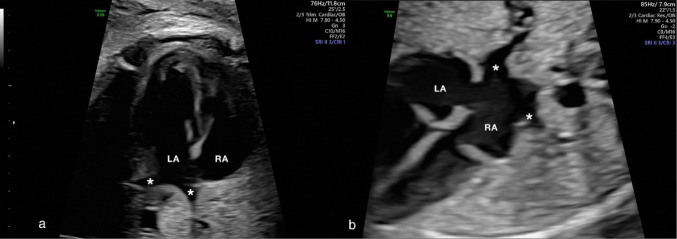


Corrected Fig. 4

The original article has been corrected.

**Figure:**